# Assessment of Health Professionals’ Attitudes on Radiation Protection Measures

**DOI:** 10.3390/ijerph182413380

**Published:** 2021-12-19

**Authors:** Aspasia Goula, Athanasios Chatzis, Maria-Aggeliki Stamouli, Martha Kelesi, Evridiki Kaba, Emmanouil Brilakis

**Affiliations:** 1 Master of Health and Social Care Management, Department of Business Administration, School of Administrative, Economics and Social Sciences, University of West Attica, 12243 Athens, Greece; hatzis21@gmail.com (A.C.); mstamouli@yahoo.com (M.-A.S.); 2 Department of Nursing, School of Health and Care Sciences, University of West Attica, 12243 Athens, Greece; mkel@uniwa.gr (M.K.); ekaba@uniwa.gr (E.K.); 3 3rd Orthopedic Department of Hygeia Hospital, 15123 Athens, Greece; Emmanuel.Brilakis@gmail.com

**Keywords:** radiation, protection, safety, knowledge, health care services, misconceptions, attitudes, perceptions

## Abstract

(1) Background: Health professionals’ knowledge, beliefs and perceptions concerning radiation protection may affect their behaviour during surgery and consequently influence the quality of health services. This study highlights the health professionals’ average knowledge level and captures the beliefs, perceptions, and behaviours in a large public Greek hospital. (2) Materials and Methods: A cross-sectional study was carried out, including health professionals working in operating rooms. One hundred thirty-two staff members participated by responding to an original questionnaire. The sample consisted of nurses, radiographers and medical doctors of various specialties involved daily in surgical procedures where ionizing radiation is required. The survey was conducted from March to June 2021, and the response rate was 97%. (3) Results: The level of overall knowledge of health professionals regarding radiation protection safety was not satisfactory. Females and employees with a lower level of education had more misconceptions about radiation and radiation protection. Employees of younger ages and with less previous experience were more likely to have negative emotions towards radiation exposure. Finally, employees with fewer children tended to express physical complaints caused by their negative emotions due to radiation exposure. (4) Conclusions: Health professionals’ lack of basic and specialized knowledge concerning radiation protection safety had a negative impact on the provision of health services. The continuing training of the staff seemed to be the only solution to reverse this trend. The training should highlight how radiation exposure can be minimized, safeguarding health professionals’ trust and sense of security by significantly improving their working environment.

## 1. Introduction

The United Nations Scientific Committee on the Effects of Atomic Radiation (UNSCEAR) notes that the general population’s exposure to ionizing radiation from natural sources is constant and cannot be avoided [1]. Ionizing radiation is known to be widely used for diagnostic and therapeutic purposes. With the advancement of medical science and the use of new practical applications over recent decades, the use of ionizing radiation has significantly increased [2,3,4,5,6]. There are two categories of ionizing radiation’s health effects. On the one hand, there are the deterministic effects that are related directly to the absorbed radiation dose and their severity increases with the dose increase. A deterministic effect typically has a threshold (of 100 mGy or higher) below which the effect does not occur. Deterministic effects are based on tissue damage. Lens opacities induced by ionising radiation and skin erythema are regarded as deterministic effects. However, the deterministic effects of ionising radiation do not need to be considered as a health hazard at the low exposures used during C-arm fluoroscopy in the surgery room. On the other hand, the stochastic effects of ionising radiation have to be considered. Stochastic effects are chance events, with the probability of the effect increasing with the received dose, but the severity of the effect is independent. Stochastic effects are assumed to have no threshold. Primarily cancer risk, but also hereditary disorders, are stochastic effects [7]. The ultimate biological effects on small amounts of radiation, although carefully studied, remain largely unknown [8,9,10,11,12,13,14,15,16,17,18,19,20,21]. Research has linked the possibility of cancer [22,23,24] and the appearance of cataracts in the eyes [25,26] after the chronic use of low-dose radiation.

Since ionizing radiation has become essential for the diagnosis and treatment of a variety of medical conditions, it is mandatory to ensure its safe use and minimize its associated risk to the patients. For this reason, all countries have regulated safety standards and established national radiation protection regulations. In Greece, the national regulatory authority competent for radiation protection, GAEC (Greek Atomic Energy Commission), has published the Greek radiation protection legislation (Presidential Decree 101/2018, Government Gazette No. 194/A/20.11.2018). Greek legislation is based on the issued European Basic Safety Standards (BSS) Directive (Directive 2013/59/Euratom) [27,28].

Most of the general population do not appear to be adequately familiar with radiation issues. One additional responsibility of health professionals is to guide and instruct individuals who are involved with ionizing radiation. The problem with this is that health professionals seem to not be familiar with this role. In a survey conducted among a thousand adults who visited a health facility to assess their knowledge concerning the potential adverse effects of radiation after a diagnostic examination, only 14.4% of them were aware of the potential risks arising from it. In the same study, only 5.2% of the participants were instructed and given radiation protection by health professionals [29]. Therefore, when entering a health facility for services involving ionizing radiation, people may have questions and concerns. It is therefore essential for health professionals, through their knowledge, to have the appropriate beliefs and perceptions of the usefulness and potential risks that may arise from radiation so that they can safely guide the patients and help them to convey the feeling of trust towards them. This may have a positive impact on the health services provided. Otherwise, health professionals cannot adequately persuade patients on the relative benefits and the possible risks involved in their diagnostic examination, and as a result, feelings of insecurity, concern, and fear, arise, leading to a poor provision of health services. This notion is in agreement with previous studies. A study among patients, emergency physicians and radiologists at a tertiary health care centre, concerning their CT radiation dose awareness and potential risks, showed that 95% of the patients had not been informed of the potential benefits and threats expected to arise after the diagnostic examination. Accordingly, 78% of emergency health professionals admitted that they had not informed the patients [30]. In another study, the percentage of patients who had not been informed about the radiation risks was 92%. In addition, 25% of the doctors working in the hospital and 43% of the medical students had misconceptions, and more specifically, were unaware that invasive procedures are performed using ionizing radiation. Another noteworthy finding was that 28% of physicians did not know that mammograms are performed using radiation [30]. Another study documented the significant differences between patients and physicians in evaluating and perceiving the health risks posed by ionizing radiation levels. The study concluded that patients’ perceptions formed a solid basis for the decisions they were called upon to make [31]. In a study conducted in a medical imaging department of a tertiary health care institution exploring its services to patients, satisfaction was high, as opposed to safety. This means that psychological and social factors may form the culture of patients’ safety. Health professionals should listen to the patients’ anxieties and worries and help them understand which are justified and not. This ensures a more efficient provision of health care [32]. All of the above indicates that there is a clear gap that should be highlighted, and this is the rationale behind this study.

The main purpose of this study was to explore the beliefs, the perceptions, and the behaviours of health professionals of a large Greek public hospital regarding the provision of health services where the use of ionizing radiation is required. Moreover, an additional objective was to research the impact of specific demographic characteristics on these beliefs.

## 2. Materials and Methods

### 2.1. Participants and Procedure

The survey was carried out at the general hospital “Asklepieio Voulas”. Quantitative cross-sectional research was conducted, using convenience sampling. This was a non-probability sampling where the sample was taken from a group of people who were easily accessible. The questionnaire was in Greek, so the primary criterion for participation in the research was that the participants could read and speak Greek fluently. It was chosen to study health professionals from various specialties (nurses, radiographers, orthopaedic surgeons, neurosurgeons, urologists, and anaesthesiologists) who are daily involved in surgical procedures where the use of ionizing radiation through C-ARM is required. According to the European and Greek Legislation, radiation exposure of, amongst others, radiation workers, is limited by law to a set of prescribed radiation exposure levels, called dose limits. The annual effective dose limit for radiation workers is 20 mSv and the annual equivalent dose is 20 mSv and 500 mSv for the lens of the eye and the skin, respectively. As part of the system of protection, those who are, or are likely to be, exposed to more than 6 mSv effective dose or more than 15 mSv equivalent eye dose or 150 equivalent skin dose are subject to additional control measures, and are classified as “Category A Workers”. The rest, who are not likely to receive the above amounts of radiation, are classified as “Category B workers”. All health professionals from the surgery department who participated in the survey were “Category B workers” except radiographers who are “category A workers”. Due to their involvement with ionizing radiation, these employees were expected to be informed, interested in, and protected from radiation. Category B workers were expected to have a higher cognitive level than the staff of other departments who do not use radiation and a lower cognitive level than Category B workers working in departments where radiation is always in use. One hundred thirty-six questionnaires were distributed, and 132 were collected. This corresponds to a response rate of 97%. The survey was conducted from March to June 2021.

All participants were provided with a written consent form, by means of a declaration, as a separate part of the questionnaire, before proceeding with the completion of the survey. Data collection guaranteed anonymity and confidentiality. All subjects were informed of their right to refuse or discontinue participation in the study, according to the ethical standards of the Helsinki Declaration.

### 2.2. Research Instrument 

The research tool used for this research was an original one [33]. It consisted of the six following sections: “General Radiation Protection Safety Knowledge” with 16 questions, “Occupational Safety and Health—Radiation Protection Safety equipment” with 17 questions, “C-arm fluoroscopy and Radiation Protection Safety” with 15 questions, “Dosimetry” with 12 questions, “Beliefs, Attitudes and Practices regarding Radiation Protection Safety” with 24, and “Demographic data” with 11 questions. Two types of validity were assessed, face and construct validities. Five leading experts evaluated face validity, who found that the questionnaire was characterized by high face validity, which means that it is clear from the questions that the instrument measures what it is designed to measure. Additionally, the final questionnaire was characterized by construct validity in each sub-dimension [33]. As regards its internal consistency, Cronbach’s Alpha correlation coefficient was used. Its values ranged between 0.499 and 0.894, and they fluctuated from acceptable to very good. At this point, it is essential to note that Cronbach’s Alpha coefficient values for dimensions E, E1, and E2 were not within the acceptable range (0.499, 0.526 and 0.511, respectively). However, they were maintained because exploratory factor analysis showed high relevance between the questions [30].

The questionnaire revealed 15 dimensions, which are the following:

A: Overall Knowledge of Radiation Protection Safety (12 questions).
A1: Basic Knowledge of Radiation Protection Safety (6 questions).
A2: Advanced Knowledge of Radiation Protection Safety (6 questions).
B: Occupational Safety and Health—Radiation Protection Safety equipment (5 questions).
C: Negative Attitude towards Radiation Protection Safety equipment (6 questions).
C1: Discomfort of wearing personal Radiation Protection Safety equipment (4 questions).
C2: Discomfort from unclean personal Radiation Protection Safety equipment (2 questions).
D: Knowledge of Dosimetry (4 questions).
E: Negative feelings due to accidental Radiation Exposure (6 questions).
E1: Fear and anger due to unintentional Radiation Exposure (4 questions).
E2: Guilt for being unintentionally exposed to radiation (2 questions).
F: Psychosomatic symptoms due to negative feelings related to radiation (4 questions).
G: Misconceptions about Radiation—Radiation Protection Safety (6 questions).
G1: Misconceptions about Radiation (4 questions).
G2: Misconceptions about the importance and necessity of Radiation Protection Safety (2 questions).

Responses to the questions that make up dimensions A, A1, and A2 are true (1)/false (0) types. These dimensions are calculated as the sum of the questions that comprise each one of them. The maximum value of dimension A is twelve if an employee correctly answers all the questions, and the minimum is 0 if an employee incorrectly answers all of them. Employees with sufficient knowledge are those who correctly answer at least six questions. This dimension can also be expressed as the sum of the dimensions A1 + A2, calculated similarly as dimension A (maximum value of each dimension is 6). Additionally, dimension D is calculated in the same way as dimension A (its maximum value is 4).

Dimensions B, C, C1, C2, E, E1, E2, F, G, G1, and G2, are calculated as mean values of the questions/variables that comprise each one of them. They correspond to questions ranging from 1 to 5 on a Likert scale, with a neutral value of 3. Values greater than 3 indicate a negative perception for dimensions C, C1, C2, E, E1, E2, F, G, G1, and G2. On the contrary, values greater than 3 indicate a positive perception for dimension B. Dimensions C, E, and G can also be calculated as mean values of dimensions (C1 and C2), (E1 and E2), and (G1 and G2).

### 2.3. Statistical Analysis

**Figure 1:**
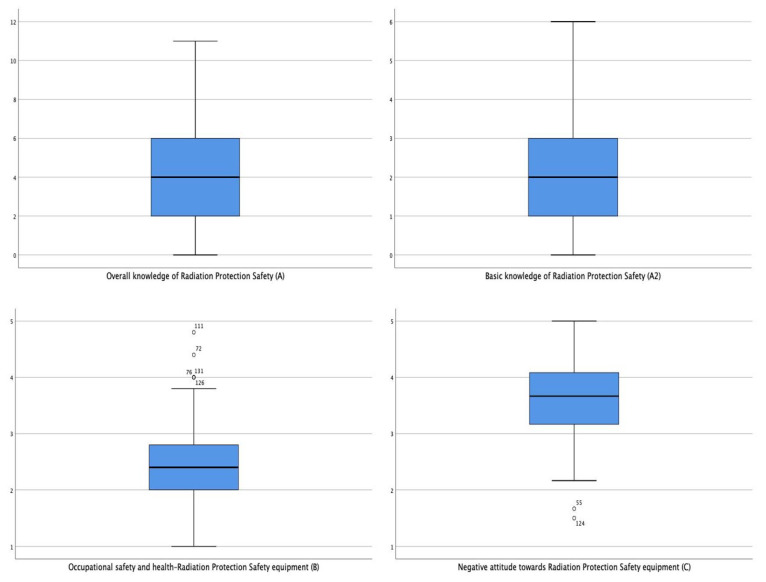

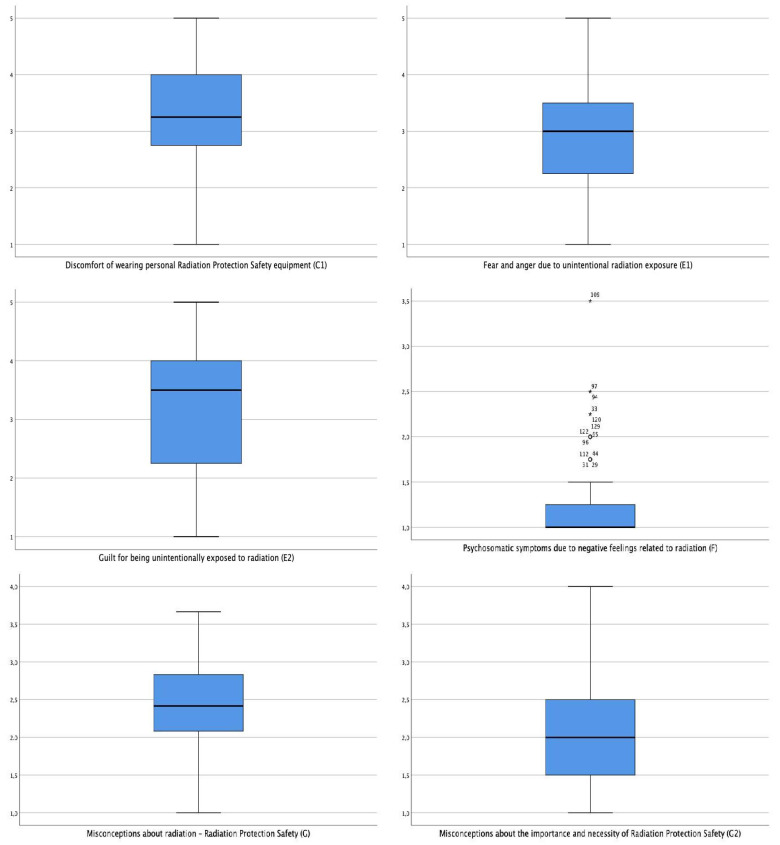
Boxplot for selected dimensions of the research tool. (**A**: Overall knowledge of radiation protection safety. **A2**: Advanced knowledge of radiation protection safety. **B**: Occupational safety and health—radiation protection safety equipment. **C**: Negative attitude towards radiation protection safety equipment. **C1**: Discomfort of wearing personal radiation protection safety equipment. **E1**: Fear and anger due to unintentional radiation exposure. **E2**: Guilt for being unintentionally exposed to radiation. **F**: Psychosomatic symptoms due to negative feelings related to radiation. **G**: Misconceptions about Radiation—radiation protection safety. **G2**: Misconceptions about the importance and necessity of radiation protection safety). stars (*) and circles (o) represent extreme and mild outliers, respectively.

Statistical analysis was carried out with SPSS version 26 ((IBM, Athens, Greece).As has already been mentioned, dimensions B, C, C1, E1, E2, F and G were calculated as mean values of the questions/variables that comprise each one of them. Dimensions A and A2 were calculated as sums of the responses to the individual questions/variables that contain them. The Kolmogorov–Smirnov test (Table 1) was applied to assess the normality of their distributions. The tests showed a statistically significant deviation from the normality for dimensions A, A2, B, C1, F, E2, F, G and G2, and their box plots revealed outliers and skewness in their distributions (Figure 1). For this reason, the statistical tests employed for their analysis were non-parametric. The distribution of dimension B, on the other hand, was not skewed. Therefore, parametric tests were used for their statistical analysis (Figure 1). Furthermore, for dimensions C and E1, the Kolmogorov–Smirnov test (Table 1) revealed no statistically significant deviation from normality, which is why the statistical tests employed were parametric.

The non-parametric one-sample Wilcoxon signed-rank test (W) and the One-sample *t*-test were used to search statistically significant differences between selected instrument dimensions and a specific value, regarded as “neutral”, or it is used to classify knowledge level as sufficient or not. Specifically, the one-sample Wilcoxon signed-rank test (W) was used for the dimensions: A (control value = 6), A1, A2, C1, C2, E, E2, F, G, G1 and G2 (control value = 3), D (control value = 2) and the one-sample *t*-test was used for the dimensions: B, C, and E1 (control value = 3).

Additionally, the non-parametric Mann–Whitney U test was used to evaluate the impact of “gender” and “number of children” on the selected dimensions of the questionnaire. Finally, the non-parametric Kruskal–Wallis H test was used to study the effect of “education level”, of “age groups”, and of “experience in years” on selected dimensions of the questionnaire. If the Kruskal–Wallis H test was significant, a post hoc analysis based on Bonferroni adjustment was conducted. The level of statistical significance was set to a = 0.05.

## 3. Results

### 3.1. Descriptive Analysis of the Sample

Most of the participants were males (54.5%). As regards their age, most of them (40.9%) were in the age group of (35–44), and in terms of education level, most of the employees (62.1%) were university graduates. Additionally, 50% of the participants stated that they have three or more children. Finally, most of the employees who participated in the research answered that they have (0–10) and (11–20) years of previous experience (31.8% and 32.6%, respectively) (Table 2).

### 3.2. Analysis of the Level of Questionnaire’s Dimensions

Tests were found to be statistically significant for the following dimensions (Table 3):

A: it was found that the observed value (value = 4.00) was statistically significantly lower than the control value (value = 6.00). This finding indicates that the level of health professionals’ comprehensive knowledge on radiation protection was not satisfactory.
A1: the test showed that the observed value (value = 2.00) was statistically significantly lower than the control value (value = 3.00). This finding indicates that the level of health professionals’ comprehensive knowledge on radiation protection was not satisfactory.
A2: it was found that the observed value (value = 2.00) was statistically significantly lower than the control value (value = 3.00). This finding shows that the level of health professionals’ specialist knowledge on radiation protection was not satisfactory.
C: it was found that the observed value (value = 3.65) was statistically significantly higher than the control value (value = 3.00), which indicates that health professionals tended to have a negative attitude towards radiation protection equipment.
C1: the test revealed that the observed value (value = 3.50) was statistically significantly higher than the control value (value = 3.00), which shows that health professionals tended to experience discomfort when they needed to wear their radiation protection equipment.
C2: the test showed that the observed value (value = 4.00) was statistically significantly higher than the control value (value = 3.00). This result indicates that health professionals did not consider the radiation protection equipment suitable for use in terms of its level of sanitation and cleanliness.
D: it was found that the observed value (value = 4.00) was statistically significantly higher than the control value (value = 2.00). This finding indicates that health professionals’ knowledge was satisfactory in terms of individual dosimeter use.
F: the test showed that the observed value (value = 1.00) was statistically significantly lower than the control value (value = 2.30). This result indicates that radiation-related negative feelings did not appear to be embodied by health professionals.
G: it was found that the observed value (value = 0.50) was statistically significantly lower than the control value (value = 3.00). This finding shows that health professionals had fewer misconceptions about radiation and radiation protection.

### 3.3. Demographic Characteristics Impact on Selected Dimensions

#### 3.3.1. Gender Factor

Based on the analysis, the Mann–Whitney-U test was used to determine the impact of the “gender” factor on the dimensions C, C1, G, and G2, the test was statistically significant for all these dimensions. Women had a higher mean rank than men on these dimensions (Table 4). 

#### 3.3.2. Education Level Factor

The non-parametric Kruskal–Wallis H test was used to evaluate the impact of the “education level” factor on dimensions A, A2, C, and G. The test was statistically significant for dimension G. According to test results, employees with a lower level of education had more misconceptions about radiation and radiation protection than employees with a higher level of education (Table 5).

#### 3.3.3. Age Groups Factor

Based on the statistical analysis with the Kruskal–Wallis H test to assess the impact of the “age groups” factor on dimensions E and F, the test was statistically significant for dimension F (Table 6).

#### 3.3.4. Previous Experience Factor

The Kruskal–Wallis H test was used to determine the impact of the “previous experience” factor on dimensions A, C, E, F, and G2 (Table 7). The test was statistically significant for the following dimensions:

A: the post hoc analysis identified statistically significant differences for the pair (11–20) vs. (21–30), where employees with less previous experience had a higher level of radiation protection knowledge.
F: the post hoc analysis which followed revealed statistically significant differences in the pairs (i) (0–10) vs. (21–30) and (ii) (11–20) vs. (21–30). That means that less experienced employees were more likely to somatise negative emotions due to radiation exposure than more experienced colleagues in both cases checked.
G2: the following post hoc analysis showed that statistically significant differences existed only for the pair (11–20) vs. (31–40), meaning that, employees with less previous experience had fewer misconceptions about the necessity for radiation protection than those with more experience.

#### 3.3.5. Number of Children Factor

The impact of the “number of children” factor on dimension F was assessed using the Mann–Whitney-U test, which proved to be statistically significant (Table 8). The test results show that when compared to individuals with more children, employees with fewer children had a higher tendency to somatise their negative emotions towards radiation.

## 4. Discussion

The most important finding of this survey was that health professionals’ cognitive level regarding radiation protection safety was not satisfactory. This lack of knowledge can lead to misconceptions and behaviours that may affect the health services provided. A series of studies seemed to confirm the findings of this research study. Son et al., in their research on the radiation protection safety of invasive case’s personnel, stated that there was a severe lack of knowledge and proposed that there was a need to heighten personnel’s awareness with adequate training [34]. In the same direction is the study of Saroki et al. concerning orthopaedic surgeons. They concluded that further training on radiation protection safety was required for orthopaedic surgeons, which should have been part of professional conferences [35]. Tok et al. found in their research that the staff of the urology surgery lacked the necessary knowledge, and consequently, the measures they were taking were not sufficient. This resulted from a lack of education, and he proposed that training in practical understanding of radiation protection should be included in their primary education [36]. The study of Brun et al. went a step further. This research revealed a lack of knowledge of anaesthesiologists and orthopaedic surgeons on radiation protection issues and limited use of radiation protective equipment. After their training, the improvement achieved in the radiation protection issues was not satisfactory. This led to the adaptation of training strategies focused on radiological risks and radiation protection safety issues [37]. Even medical radiologists and radiographers fall short in terms of sensitivity on radiation protection safety and procedures for which ionising radiation is used, according to the study of Paolicchi et al. That implies the necessity of regular education and training courses. It is worth mentioning that only 12.1% of the staff, according to the same study, attended radiation protection courses regularly [38]. On the contrary, the Park et al.’s survey of 129 nurses from emergency departments investigating the factors that influence behaviours around radiation protection a positive correlation found to be statistically significant between knowledge about radiation protection and behaviours on radiation protection issues. Being more aware of radiation protection was associated with better performance in the behaviours around radiation protection, affecting the quality in the health services provided [39].

In some cases, the negative behaviour of health professionals is due to objective conditions such as, for example, a negative attitude towards radiation protective equipment. The present research has shown that employees are negative in using radiation protection because they are heavy, dirty, and smell bad, and when forced to wear them, they resent it. Klein et al. expressed that lead aprons undoubtedly have many advantages for personal radiation protection. However, their weight and size are such that they can cause musculoskeletal damages mainly to the spine [40]. According to Goldstein et al., in research conducted by interventional cardiologists to investigate the possibility of orthopaedic problems caused by lead aprons, 42% responded that they had problems with the spine and 28% with other joints (i.e., hip, knee). The problems were significant in some cases since they were absent from their work for days [41]. 

In addition, usually, not all the appropriate sizes that correspond to all body types are available. Thus, when wearing their equipment, overweight employees feel trapped, while for the thin ones, the equipment is also problematic due to its large size. Although research on style and size is of limited range, Cremen et al., while investigating surgeons’ exposure to interventional radiology, concluded that the use of unfit radioprotective equipment in terms of its size can have undesirable effects on its radioprotective competence and can cause discomfort to staff. Lead aprons that are very big concerning the body type of the employee may allow the scattered radiation to reach the chest through the large holes in the shoulder girdle’s area. They are also likely to cause musculoskeletal problems due to their increased weight. Additionally, too small-sized radioprotective equipment may not adequately cover all body parts that must not remain exposed during X-ray examination [42]. A possible answer to the musculoskeletal problems of the personnel could be the personal equipment of radiation protection that does not have lead or that use complex shielding materials (lead with cadmium, with iodine, or with tin) and are lighter. This is an ideal situation but has significant difficulties in implementation. Some studies have shown that such radioprotective means exhibit the same or even better properties in terms of radiation’s attenuation than classic lead aprons [43].

Staff’s refusal to use the equipment may also be because they do not know if the means of personal radiation protection are checked for their suitability (cracks in the lead, cracks in the aprons and collars). The wrong way of storing the equipment (e.g., several aprons stacked one on top of the other on the same hanger, collars and aprons thrown on the floor) enhances the negative attitude of employees. In a study carried out by Mohd Ridzwan et al. concerning staff’s beliefs about radiation protective equipment, it was deemed necessary to have quality control on the equipment of personal radiation protection to be able to ascertain safely that they are suitable and that they absorb radiation following their specifications [44]. Negative perceptions are also due to the lack of knowledge about the crucial role of the radiation protective personal equipment in protecting the health professionals from ionising radiation. It is worth mentioning that the effectiveness of lead aprons has been evaluated in many studies until today. Most of them report that lead aprons and collars of the thyroid gland, 0.25 mm and 0.50 mm thick, prevent more than 90% and 98% of the radiation, respectively [45,46,47]. Adequacy of equipment for all staff and in all sizes, as well as regular biannual check for its suitability followed by a written report of the way it was checked and the results, combined with targeted training on the practical aspects of applying for radiation protection, would help to halt these attitudes/perceptions.

This study also found that the employees knew to a satisfactory degree that the personal dosimeter serves to record the dose that someone involved with radiation is likely to receive. However, some respondents expressed doubt about whether the dosimeter’s recording and measurement of radiation were carried out correctly. This doubt may create insecurity and negative emotions.

The survey results showed that, although health professionals did not have physical complaints caused by their negative emotions towards radiation, there were categories of the demographic characteristics’ results for which this was not the case. More specifically, physiological resonance was inversely proportional to previous experience. The shorter the employee’s previous experience, the greater the tendency to exhibit stress symptoms (pain in the eyes, burning sensation, sickness) due to radiation. The same appeared in the age category too, where younger people (35–44) somatised the anxiety more than the older ones (55+). That means younger employees were more likely to have physical symptoms due to negative emotions because of radiation exposure than older employees.

In addition, employees with one to two children experienced anxiety events to a greater extent than those with more children. The main factor that led to a stressful event was the lack of knowledge and training in protection from radiation. In research carried out by Alavi et al., employees with low performance in tests carried out for their cognitive level in radiation protection, their perceptions and practical training showed work-related stress to a greater extent than their colleagues who performed better on the tests [48]. In the same direction are other studies showing that adequate training, positive perceptions leading to positive behaviours, and good practices reduce stressful events among staff [49,50,51].

The present research has shown that, in general, health professionals had minor misconceptions concerning radiation and the need for radiation protection. Where gender was involved, the results showed that women were generally unfavourable to radiation protection equipment and their discomfort was heightened when required to wear it. Furthermore, women appeared to have more misconceptions about radiation and more misconceptions about the importance and the necessity of radiation protection safety than men.

It is also the case with employees of a lower level of education compared to those of a higher level of education. The same applied to those with work experience of 11 to 20 years compared to those working 31 to 40 years. In this case, less experience equals more misconceptions.

Analysing the results, the effect of gender on misconceptions about radiation and radiation protection may be caused by the fear of having children and the stress created by continuous fluoroscopy. These results arise from specific variables found in the questionnaire and refer more specifically to the possibility of not having children due to radiation and the stress caused to the employee by continuous fluoroscopy in the operating room. 

Concerning the effect of previous experience on misconceptions, in their study, Seifi et al., after dividing employees into two categories, one with work experience of up to 15 years and the other with more than 15 years of experience, found that employees with up to 15 years of experience had less knowledge of the undesirable effects of radiation and this probably led to misconceptions [52]. This finding is consistent with earlier research by Mohiri et al., according to which employees with less work experience had correspondingly less knowledge of the undesirable effects of radiation [53]. In addition, another study, according to which participants who had less than ten years of employment had a poor level of knowledge of radiation protection safety aspects, which resulted in an increased risk of undesirable situations [54].

Regarding the impact of educational level, the results revealed that employees with a higher degree had fewer misconceptions. Alavi et al. also found out that educated radiographers knew about the potential risks from radiation exposure and they exhibited more professional behaviour concerning their work [55]. Their training on radiation and radiation protection safety issues allowed them to have a dominant role since, as qualified professionals with their knowledge and they could provide quality health services. The same research emphasized that a high level of studies is a crucial factor for job satisfaction. More specifically, obtaining a higher education degree creates a feeling of self-confidence, personal development, self-realization, and success at work. Combining these factors, the staff acquires better professional satisfaction, an essential ingredient for providing quality health services [52].

The strength of this study is that it calculated the beliefs, perceptions and behaviours of health professionals using a prototype and original measuring tool, explicitly constructed for serving this aim, trying to correlate the knowledge of the specialized personnel with the quality of the service provided. The study’s hospital is one of Greece’s high volume public institutions and reflects a representative sample of a typical public hospital in our country.

### Limitations of Study

This study also had some limitations. Firstly, the findings of the survey referred to one general hospital in Attica, so the results can only be generalized to this hospital. Further data collection from different size hospitals will be more reliable, reflecting health professionals’ attitudes on radiation protection measures. Secondly, qualitative research through observation would try to understand the behaviour, experience, intentions and motivations of health professionals around the subject under study, would be very useful. 

## 5. Conclusions

Health professionals’ lack of basic and specialized knowledge concerning radiation protection safety has a negative impact on the provided health services. The continuing training of staff seems to be the only solution to reverse this trend. Educational training seminars should provide adequate knowledge about all the important issues concerning radiation protection, emphasizing the staff radiation exposure, the related radiation risk, the importance of radiation safety equipment, and the practical implementation of theoretical safety knowledge. The training should highlight how radiation exposure can be minimized, safeguarding their trust and sense of security, and significantly improving their working environment, while keeping in mind the core radiation protection principles: (i) the principle of justification; (ii) the principle of protection optimization; and (iii) the principle of dose limit application [56]. All the above can reverse the negative attitude that a health professional may have, which can surely improve the services provided.

## Figures and Tables

**Table 1:** Normality test for selected dimensions of the research tool.

	Kolmogorov–Smirnov ^a^		
	Statistic	Df	Sig.
A *	0.144	116	<0.001
A2 *	0.167	116	<0.001
Β *	0.126	116	<0.001
C *	0.066	116	0.200
C1 *	0.098	116	0.008
Ε1 *	0.078	116	0.078
Ε2 *	0.189	116	<0.001
F *	0.415	116	<0.001
G *	0.097	116	0.009
G2 *	0.133	116	<0.001

^a^ Lilliefors Significance Correction. * A: Overall knowledge of radiation protection safety. A2: Advanced knowledge of radiation protection safety. B: Occupational safety and health—radiation protection safety equipment. C: Negative attitude towards radiation protection safety equipment. C1: Discomfort of wearing personal radiation protection safety equipment. E1: Fear and anger due to unintentional radiation exposure. E2: Guilt for being unintentionally exposed to radiation. F: Psychosomatic symptoms due to negative feelings related to radiation. G: Misconceptions about Radiation—radiation protection safety. G2: Misconceptions about the importance and necessity of radiation protection safety.

**Table 2:** Sampling frame description.

		Frequency	Percent (%)
Gender	Male	72	54.5
	Female	60	45.5
Age Group	25–34	17	12.9
	35–44	54	40.9
	45–54	45	34.1
	55+	16	12.1
Education Level	Secondary Education	29	22.0
	Technological Education	21	15.9
	University Education	82	62.1
Number of Children	1–2	64	48.5
	2+	66	50.0
	Missing Values	2	1.5
Experience (in years)	0–10	42	31.8
	11–20	43	32.6
	21–30	29	22.0
	31–40	18	13.6
Total		132	100.0

**Table 3:** Results of one-sample Wilcoxon signed-rank test and one-sample *t*-test for selected dimensions of the research instrument.

Dimensions	Test Value	Observed Value	Test *p*-Value
A *	6.00	4.00	W = 931.50 *p* < 0.001 **
A1 *	3.00	2.00	W = 1364.50 *p* < 0.001 **
A2 *	3.00	2.00	W = 611.00 *p* < 0.001 **
B *	3.00	2.41	t (131) = −9.525 *p* < 0.001 **
C *	3.00	3.65	t (131) =9.796 *p* < 0.001 **
C1 *	3.00	3.50	W = 5508.00 *p* < 0.001 **
C2 *	3.00	4.00	W = 6936.00 *p* < 0.001
D *	2.00	4.00	W = 6973.00 *p* < 0.001 **
E *	3.00	3.00	W = 3736.50 *p* = 0.729
Ε1 *	3.00	2.89	t(131) = −1.231 *p* = 0.220
Ε2 *	3.00	3.50	W = 4093.50 *p* = 0.292
F *	3.00	1.00	W = 4.50 *p* < 0.001 **
G *	3.00	2.50	W = 432.50 *p* < 0.001 **

* A: Overall knowledge of Radiation Protection Safety. A1: Basic knowledge of radiation protection safety. A2: Advanced knowledge of radiation protection safety. B: Occupational safety and health—radiation protection safety equipment. C: Negative attitude towards radiation protection safety equipment. C1: Discomfort of wearing personal radiation protection safety equipment. C2: Discomfort from unclean personal radiation protection safety equipment. D: Knowledge of dosimetry. E: Negative feelings due to accidental radiation exposure. E1: Fear and anger due to unintentional radiation exposure. E2: Guilt for being unintentionally exposed to radiation. F: Psychosomatic symptoms due to negative feelings related to radiation. G: Misconceptions about radiation—radiation protection safety. ** test significant at 0.01 level.

**Table 4:** Mann–Whitney-U test results for the evaluation of the impact of gender on selected dimensions.

Dimensions	Gender	N	Mean Rank	Test *p*-Value
C *	Male	72	58.43	U = 1579.00 *p* = 0.008 **
	Female	60	76.18	
C1 *	Male	72	58.18	U = 1561.00 *p* = 0.006 **
	Female	60	76.48	
G *	Male	72	56.45	U = 1436.50 *p* = 0.001 **
	Female	60	78.56	
G2 *	Male	72	57.68	U = 1525.00 *p* = 0.003 **
	Female	60	77.08	

* C: Negative attitude towards radiation protection safety equipment. C1: Discomfort of wearing personal radiation protection safety equipment. G: Misconceptions about radiation—radiation protection. G2: Misconceptions about the importance and necessity of radiation protection safety. ** test significant at 0.01 level.

**Table 5:** Kruskal–Wallis H test results for the evaluation of Education Level impact on selected dimensions.

Dimensions	Level of Education	N	Mean Rank	Test *p*-Value	Post hoc Analysis
A *	Secondary education	26	57.35	H = 1.308 *p* = 0.520	-
	Technological education	21	64.48		
	University education	81	66.80		
A2 *	Secondary education	20	62.28	H = 0.524 *p* = 0.769	-
	Technological education	20	55.25		
	University education	76	58.26		
C *	Secondary education	28	75.91	H = 2.863 *p* = 0.239	-
	Technological education	21	68.10		
	University education	82	62.08		
G *	Secondary education	28	88.18	H = 12.458 *p* = 0.002	test statistic = 28.965 *p* = 0.001 **
	Technological education	21	62.93		
	University education	82	5.21		

* A: Overall knowledge of radiation protection. A2: Advanced knowledge of radiation protection safety. C: Negative attitude towards radiation protection safety equipment. G: Misconceptions about radiation—radiation protection safety. ** test significant at 0.01 level.

**Table 6:** Kruskal–Wallis H test results for the evaluation of age group impact on selected dimensions.

Dimensions	Age Group	N	Mean Rank	Test *p*-Value	Post hoc Analysis
Ε	25–34	17	53.29	H = 3.651 *p* = 0.302	-
	35–44	54	72.69		
	45–54	45	65.46		
	55+	16	62.59		
F	25–34	17	75.38	H = 14.348 *p* = 0.002	test statistic = 27.139 *p* = 0.001 (35–44) − (55 + ) **
	35–44	54	75.14		
	45–54	45	59.36		
	55+	16	48.00		

E: Negative feelings due to accidental radiation. F: Psychosomatic symptoms due to negative feelings related to radiation. ** test significant at 0.01 level.

**Table 7:** Kruskal–Wallis H test results for the evaluation of years of experience impact on selected dimensions.

Dimensions	Years of Experience	N	Mean Rank	Test *p*-Value	Post hoc Analysis
A *	0–10	41	64.32	H = 8.478 *p* = 0.037	test statistic = 24.937 *p* = 0.029 (11–20) − (21–30) **
	11–20	42	73.38		
	21–30	29	48.45		
	31–40	16	70.75		
	11–20	39	66.44		
	21–30	25	45.82		
	31–40	13	74.38		
C *	0–10	42	66.24	H = 3.313 *p* = 0.958	-
	11–20	43	65.31		
	21–30	29	69.84		
	31–40	18	64.56		
Ε *	0–10	42	56,23	H = 7.042 *p* = 0.071	-
	11–20	43	75.85		
	21–30	29	61.91		
	31–40	18	75.53		
F *	0–10	42	72.74	H = 13.784 *p* = 0.003	test statistic = 22.273 *p* = 0.033 (0–10) − (21–30) ** test statistic = 22.232 *p* = 0.013 (11–20) − (21–30) **
	11–20	43	74.70		
	21–30	29	52.47		
	31–40	18	54.97		
G2 *	0–10	42	71.80	H = 8.395 *p* = 0.039	test statistic = −27.913 *p* = 0.049 (11–20) − (31–40) **
	11–20	43	54.70		
	21–30	29	66.33		
	31–40	18	82.61		

* A: Overall knowledge of radiation protection safety. C: Negative attitude towards radiation protection safety equipment. E: Negative feelings due to accidental radiation exposure. F: Psychosomatic symptoms due to negative feelings related to. G2: Misconceptions about the importance and necessity of radiation protection safety. ** test significant at 0.05 level.

**Table 8:** Mann–Whitney-U test results for the evaluation of the impact of the number of children on the F dimension.

Dimensions	Number of Children	N	Mean Rank	Test *p*-Value
F *	1–2	64	71.98	U = 1697.00 *p* = 0.013 **
	3+	66	59.21	

* F: Psychosomatic symptoms due to negative feelings related to radiation. ** test significant at 0.05 level.

## Data Availability

Restrictions apply to the availability of these data. Data were collected from Post-Graduate Program “Health and Social Care Management” of the Department of Business Administration of the University of West Attica and are available from Goula A. with the permission of MSc Program.
